# Psychometric properties of two physical activity questionnaires, the AQuAA and the PASE, in cancer patients

**DOI:** 10.1186/1471-2288-11-30

**Published:** 2011-03-16

**Authors:** Roberto DK Liu, Laurien M Buffart, Marie José Kersten, Marjolein Spiering, Johannes Brug, Willem van Mechelen, Mai JM Chinapaw

**Affiliations:** 1 EMGO Institute for Health and Care Research, Department of Public and Occupational Health, VU University Medical Center, Amsterdam, the Netherlands; 2 EMGO Institute for Health and Care Research, Department of Epidemiology and Biostatistics, VU University Medical Center Amsterdam, the Netherlands; 3 Department of Hematology, Academic Medical Center, Amsterdam, the Netherlands

## Abstract

**Background:**

This study aimed to evaluate the reliability and validity of two self-report physical activity (PA) questionnaires - the AQuAA (Activity Questionnaire for Adults and Adolescents) and PASE (Physical Activity Scale for the Elderly) - in cancer patients.

**Methods:**

Test-retest reliability was determined by administering the questionnaires twice within 5 days. Intraclass correlation coefficient (ICC), standard error of measurement (SEM) and smallest detectable difference (SDD) were calculated. Construct validity was determined by comparing the questionnaire results with ActiGraph accelerometer scores using Spearman correlation coefficients (r_s_) and ICCs. Content validity was examined using the Three-Step Test-Interview (TSTI).

**Results:**

Reliability for the AQuAA scores were fair to excellent (ICC = 0.57 to 0.78). Reliability for the PASE scores ranged from good to excellent (ICC = 0.67 to 0.90). Correlations between the ActiGraph and the AQuAA and the PASE were low (r_s _= 0.05 and 0.16 respectively, and ICC = -0.001 to 0.44). The TSTI showed that participants experienced difficulties with the examples provided with the questions, the perceptions of intensity level of PA, and with recalling the time spent on PA.

**Conclusions:**

Both questionnaires showed good to excellent test-retest reliability for most scores. Construct validity of both questionnaires was low, as indicated by the low correlations with the ActiGraph. Except for a few difficulties that participants perceived when filling out the questionnaires, the content validity of both questionnaires was good.

## Background

Advances in early cancer detection and treatment strategies, have led to increased survival rates of people diagnosed with cancer [1]. Currently the overall 5-year survival rate in the Netherlands is 56% for men and 62% for women [2]. However, cancer and its treatment are associated with considerable long-term psychosocial and physical symptoms, including an increased risk of developing anxiety and depression, reduced physical fitness and cancer-related fatigue [3,4]. This may negatively impact a patient's quality of life (QoL) [3]. Recent evidence suggests that physical activity (PA) may improve the QoL of cancer patients and survivors, and higher PA levels have been associated with improved survival [5-7]. Improving PA levels may therefore be an important part of cancer rehabilitation.

To assess PA levels in cancer patients, valid and reliable assessment measures are needed. In general, questionnaires are an easy, acceptable and relatively inexpensive method to assess PA levels in large study populations [8]. Previously used questionnaires to evaluate PA levels in cancer patients are the Godin Leisure Time Exercise Questionnaire (GLTEQ) [9], the 7-day Physical Activity Recall (PAR) [10] and the PA measure of the Women's Health Initiative (WHI) [11]. Since measurement properties differ between study populations and settings, the PA questionnaire should be proven valid and reliable in the population of interest [12]. To date only few studies have examined the reliability and validity of PA questionnaires in cancer patients. One study showed that the PAR had superior validity compared to the International Physical Activity Questionnaire (IPAQ) [13]. Another study found comparable validity between the PA measure of the Women's Health Initiative PA questionnaire (WHI) and the PAR [14].

However, the GLTEQ, PAR and WHI have several shortcomings: (a) the GLTEQ estimates leisure-time exercise only and does not take into account other relevant daily activities such as household and work-related activities, (b) the PAR and the WHI focus on moderate to very hard intensity PA, thereby disregarding light intensity activities such as household chores and light leisure time exercise, and (c) the WHI assesses PA over the past month [14] making this questionnaire probably less suitable to determine the effect of exercise interventions, which usually have a time frame of around 12 weeks [15,16].

The Activity Questionnaire for Adults and Adolescents (AQuAA) [17] and the Physical Activity Scale for the Elderly (PASE) [18] are PA questionnaires taking into account leisure time, household and work-related activities of various intensity levels and recall PA over the past week. The reliability and validity of these questionnaires have been established in the general population [17,18], and the PASE has previously been used to evaluate exercise interventions in cancer patients [19,20]. However, the psychometric properties of these questionnaires among cancer patients are unknown. Therefore the present study aims to establish the test-retest reliability and the validity of the AQuAA and PASE in cancer patients.

## Methods

### Study Sample

Patients were recruited from the departments of Hematology, Oncology, Radiotherapy and Gynaecology of Academic Medical Centre (AMC) and the VU University Medical Center (VUmc) in Amsterdam from January to April 2010. The eligibility criteria were: (a) histologically confirmed primary cancer, treated with (neo adjuvant) chemotherapy, or histologically confirmed (relapsed) hematologic malignancy with no indication of progressive disease, treated with high-dose chemotherapy followed by autologous stem cell transplantation; (b) age between 18 and 70 years; (c) having received the last (active) treatment within 1 year prior to participation into this study, and (d) World Health Organization (WHO) performance status of 0 (asymptomatic; fully active and ambulatory) or 1 (symptomatic but completely ambulatory; restricted in physically strenuous activities but able to carry out light and sedentary activities). Patients who received chemotherapy only as palliative treatment were excluded from participation. The study protocol was approved by the Medical Ethics Committee of both the AMC and VUmc. Patients signed an informed consent statement before participating in the study.

A total of 53 out of 105 eligible patients, response rate 50%, agreed to participate in the study. Three of them withdrew from the study due to time constraints. There was no difference between the participants and non-responders in age (*p *= 0.90) as tested with an independent T-test, and gender (*p *= 0.57) and type of diagnosis (*p *= 0.11) as tested with a Chi-Square test. The characteristics of the responders and non-responders are presented in Table 1.

**Table 1:** Characteristics of the responders and non-responders (n = 102).

Age (y)	Responders (n = 50)	Non-responders (n = 52)	Difference
Mean (standard deviation)	50 (12)	52 (12)	
Range	23 - 68	19 - 69	
T-test			*p *= 0.90
			
**Sex, n (%)**			

Male	23 (46)	21 (40)	
Female	27 (54)	31 (60)	
Chi-square test			*p *= 0.57
			
**Diagnosis, n(%)**			

*Solid tumors*	19 (38)	34 (65)	
Ovarian cancer	2 (4)	3 (9)	
Breast cancer	4 (8)	8 (24)	
Colorectal cancer	3 (6)	9 (26)	
Cervical cancer	7 (14)	6 (18)	
Other	3 (6)	8 (24)	
			
*Hematologic malignancies*	31 (62)	18 (35)	
Multiple myeloma	5 (10)	3 (17)	
Non-Hodgkin's lymphoma	19 (38)	8 (44)	
Hodgkin's lymphoma	7 (14)	7 (39)	
Chi-square test			*p *= 0.11
			

**Treatment, n (%)**			
Chemotherapy	50 (100)		
+ Autologous Stem Cell Transplantation	15 (30)		
+ Radiotherapy	10 (20)		
+ Surgery	2 (4)		
+ Radiotherapy + Surgery	1 (1)		

### Procedures

**Figure 1:**
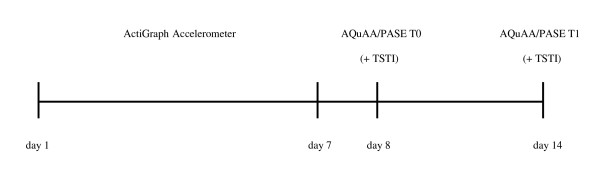
**Schematic representation of the study**. AQuAA: Activity Questionnaire for Adults and Adolescents; PASE: Physical Activity Scale for the Elderly; TSTI: Three-Step Test Interview.

Participants were fitted with an accelerometer for 7 consecutive days, see Figure 1. On the 8^th ^day they completed both the AQuAA and the PASE at their homes (T0). Five days later the questionnaires were completed for a second time (T1). No specific instructions were given with regard to the order in which the questionnaires were to be completed. At T0 or T1, we conducted a Three-Step Test-Interview (TSTI) in a subpopulation. The validity and the reliability study involved the same participants. We aimed to enrol 50 participants into the study since this is considered an adequate sample size for studies assessing the validity and reliability of measurement instruments [12].

### Activity Questionnaire for Adults and Adolescents (AQuAA)

The AQuAA is a short, self-report 7-day PA recall questionnaire [17], designed to assess daily PA and sedentary behaviour in adults and adolescents. It is divided into five categories; commuting activities, PA at work/school, household chores, leisure time activities and active sports. Each activity can be complemented with its frequency (number of days in the last week), duration (hours, minutes) and the perceived intensity (low, medium or high). Five main outcomes can be calculated: a total PA score (the AQuAA score in Metabolic Equivalent of Tasks (MET)*min/week, including all activities ≥ 2 METs), and the total time (in min/week) spent on sedentary (< 2 MET), light (2-4 METs), moderate (4-6.5 METs) and vigorous (> 6.5 METs) intensity activities. In healthy adults, test-retest reliability was fair to moderate (Intraclass correlation coefficients (ICCs) = 0.49 to 0.60) on all outcomes except for time for time spent on vigorous activities (ICC=-0.005) [17], and the correlations with the ActiGraph accelerometer were low and not significant (r_s_=-0.16 to 0.15) [17].

### Physical Activity Scale for the Elderly (PASE)

The PASE is a brief, self administered 7-day recall questionnaire specifically designed to assess PA in older adults [18]. The PASE consists of questions on leisure time, household and work-related activities. The frequency of these activities are recorded as never, seldom (1-2 days/week), sometimes (3-4 days/week), or often (5-7 days/week). The duration of activities is categorized as less than 1 hour, between 1 and 2 hours, between 2 and 4 hours, or more than 4 hours. Paid or volunteer work, except for work that involves mostly sitting activities such as office work, is categorized as less than 1 hour, between 1 and 4 hours, between 5 and 8 hours, or more than 8 hours [21]. The total PASE sum score is computed by multiplying the amount of time spent on each activity (in hours/week) by the empirically derived item weights and summing over all activities. In healthy elderly the PASE has been shown to have high test-retest reliability (r_p _= 0.84) [18] and reasonable validity as compared with the doubly labelled water method (r_s _= 0.68) [22].

### ActiGraph Accelerometer

PA was objectively assessed using the ActiGraph accelerometer (type ActiTrainer, Manufacturing Technology Inc., Pensacola, FL), a small (8.5 × 3.5 × 1.5 cm) and lightweight (51 g) PA monitor. The accelerometers were initialized according to the manufacturer's specifications, and PA was recorded in epoch intervals of 15 seconds. The participants were instructed to wear the accelerometer for seven consecutive days on their right hip during all waking hours. Since the accelerometer is not waterproof, it was not worn during water-based activities. A simple journal was provided to register the time of waking up and going to sleep, and any other instances during the day at which the accelerometer was not worn. We converted the uni-axial vertical accelerations measured by the ActiGraph into activity counts per minute. The Freedson regression-based equation was applied to categorize the activities into sedentary (< 100 counts/min), and light (< 3.0 METs or 100-1951 counts/min), moderate (3.0-5.9 METs or 1952-5724 counts/min) and vigorous (≥ 6.0 METs or ≥ 5725 counts/min) intensity [23]. A wearing day was considered valid if data was collected for at least 600 minutes (10h) that day. Non-wearing time was defined as 60 minutes of consecutive zero counts [24]. Data collected for at least 5 of the 7 wearing days were included in the validity analysis. All accelerometer data were analysed using the MeterPlus Version 4.2 software from Santech, Inc. http://www.santechhealth.com. Accelerometry has been shown to be a reasonably valid method to objectively assess PA in adults [17].

### Test-retest Reliability

Reliability concerns the degree to which a measurement is free from measurement error [25]. The test-retest reliability of the AQuAA and the PASE was assessed by the extent to which repeated administrations of the instruments in the same subjects and under the same circumstances provided similar results. Since both PA questionnaires had a recall period of 7 days, a time interval of 5 days between repeated measurements was considered appropriate to be short enough to avoid (clinical) changes in PA levels and long enough to prevent recall bias [26].

ICCs were calculated by dividing the variance between patients by the total variance [27]. An ICC value less than 0.40 was rated as poor, 0.40-0.59 as fair, 0.60-0.74 as good and values exceeding 0.75 as excellent [28]. The standard error of measurement (SEM) was calculated by taking the square root of the error variance. The corresponding smallest detectable difference at a 95% confidence level (SDD_95_) was calculated using the following formula: 1.96 × √2 × SEM [26].

In order to compare the SDD of the AQuAA and the PASE, SDDs were expressed as percentage of the measurement range [29]. To exclude potential outliers, we determined the range (range_95_) by the differences between the lowest (2.5^th ^percentile) and highest (97.5^th ^percentile) observed values for the different measures. The measurement error of an instrument may be considered small enough when the instrument is able to distinguish 7 steps (with a range from 5 to 9) on the measurement range [30]. Therefore, we considered a questionnaire with a SDD_95_/range_95 _ratio ≤ 0.20 to be useful for clinical practice [29].

### Construct Validity

Construct validity concerns the degree to which an instrument truly measures the construct it claims to measure [12]. Since there is no gold standard for measuring PA, we assessed the construct validity by comparing the AQuAA and the PASE scores with data from the ActiGraph accelerometer [12].

The data were checked for normality using normal probability plots and the Kolmogorov-Smirnov test. Since data were not normally distributed, we calculated Spearman correlation coefficients (r_s_) between total activity counts of the ActiGraph accelerometer (counts/min) and total scores of the AQuAA (MET*min/wk) and the PASE (PASE score).

ICCs between the ActiGraph accelerometer, and respectively the AQuAA and the PASE, were calculated for time spent on total physical and sedentary activities (expressed in min/wk). For the AQuAA, additional ICCs were calculated for time (in min/wk) spent on light and moderate-to-vigorous intensity activities. Based on systematic reviews on psychometric properties of PA questionnaires [31,32], we considered an r ≥ 0.50 as adequate.

### Content Validity

Content validity addresses the degree to which an instrument's content adequately reflects the construct to be measured [12]. We used the Three-Step Test Interview (TSTI), an observation-based procedure to identify response problems in self-administered questionnaires [33], to analyse how participants interpreted and responded to the questions. The interview consisted of three consecutive steps; (1) *concurrent think aloud*, aimed at collecting observational data, (2) *focused interview*, aimed at remedying gaps in observational data and (3) *semi-structured interview*, aimed at eliciting experiences and opinions with regard to the questionnaire. The TSTI is a validated pre-testing tool [34] and has previously been used to analyse cognitive processes in cancer patients [35]. Interviews were conducted until "theoretical saturation" was reached, i.e. no new information emerged [34].

The interviews were conducted either at the participants' homes, work or hospital, in accordance with the participants' preferences. Before the interview began, the steps and procedures of the TSTI were explained to the participants. The participants answered both questionnaires, and the order was alternated between subsequent interviews. To further decrease potential bias from answering the first questionnaire, the participants were instructed not to refer to the answers they provided with the first questionnaire while answering the second one.

We constructed three themes with associated questions to identify the participants' opinions of the questionnaires; "Interpretability" ("*Is the phrasing of the questionnaires clear to you?*"), "Comprehension" ("*Are all (of your) daily physical activities addressed in the questionnaires?*") and "Preference" ("*Which questionnaire were you more comfortable with answering?*"). All interviews were audio-taped and transcribed verbatim. A summary of each individual participant's statements regarding the different themes was made and typical verbatim quotations of commonly shared statements were reported.

## Results

### Test-retest Reliability

Table 2 presents median total scores and time spent on physical and sedentary activities during test (T0) and retest (T1), together with ICCs (95% confidence intervals (CI)), SEM, SDD_95 _and SDD_95_/range_95 _ratios.

**Table 2:** Test-retest reliability of the AQuAA and the PASE scores.

Instrument	T0 Median (25;75th percentile)	T1 Median (25;75th percentile)	ICC (95% CI)	SEM	SDD_95_	SDD_95_/range_95 _ratio
**AQuAA (n = 50)**						
AQuAA Score (MET*min/wk)	5316 (3263;8317)	5217 (3953;7491)	0.70 (0.53 to 0.82)	2671	7405	0.39
Total physical activities (min/wk)	1330 (913; 2321)	1500 (1054;2069)	0.63 (0.42 to 0.77)	663	1838	0.44
Light-intensity activities (min/wk)	1063 (660; 1853)	1215 (683;1706)	0.57 (0.35 to 0.73)	596	1653	0.40
Moderate-to-vigorous intensity activities (min/wk)	230 (34; 450)	293 (60;481)	0.70 (0.52 to 0.82)	275	762	0.39
Sedentary activities (min/wk)	3090 (2078; 3720)	2743 (1841; 3709)	0.78 (0.65 to 0.87)	637	1767	0.30
**PASE (n = 50)**						
PASE Sum score (PASEscore)	86 (49;161)	97 (55;175)	0.89 (0.82 to 0.94)	30	84	0.22
Total physical activities (min/wk)	1518 (873; 2630)	1659 (1005;2904)	0.90 (0.83 to 0.94)	498	1379	0.15
Sedentary activities (min/wk)	1079 (542;1802)	1079 (542;1802)	0.67 (0.48 to 0.80)	335	928	0.79

AQuAA: Activity Questionnaire for Adults and Adolescents; CI: confidence interval; ICC: intraclass correlation coefficients; PASE: Physical Activity Scale for the Elderly; SEM: standard error of measurement; T0: the first time the questionnaires were completed; T1: the second time the questionnaires were completed (5 days after the first time); SDD_95_: smallest detectable difference at a 95% confidence level; range_95_: the difference between the lowest (2.5^th ^percentile) and highest (97.5^th ^percentile) observed value.

Test-retest reliability of the AQuAA score was good; ICC = 0.70. The SDD_95_/range_95 _ratios ranged from 0.30 to 0.44, indicating that the AQuAA can distinguish 2 to 3 steps on the observed measurement range.

The ICCs for the PASE were good to excellent (ICC = 0.67 to 0.90. The SDD_95_/range_95 _ratios for the PASE were 0.22, 0.15 and 0.79, indicating that the PASE can distinguish 5 steps on the observed measurement range for the PASE sum score, 7 steps for the time spent on total PA and 1 step for the time spent on sedentary activities.

### Construct Validity

Two participants reported inconveniences with wearing the accelerometer and consequently did not have enough wearing days to be able to assess their daily PA. Therefore, they were excluded from the validity analyses. Table 3 presents the correlation coefficients between the AQuAA, the PASE and the ActiGraph data.

**Table 3:** Construct validity of the AQuAA and the PASE scores.

Instrument	Median (25;75th percentile)	Correlation with the ActiGraph accelerometer		
		r_s_	*p*	ICC (95% CI)
**ActiGraph (n = 48)**				
Counts/min	289 (195;337)			
Total physical activities (min/wk)	1348 (1117;1717)			
Light-intensity activities (min/wk)	1120 (935;1448)			
Moderate-to-vigorous intensity activities (min/wk)	223 (132;316)			
Sedentary activities (min/wk)	4329 (3551;4584)			
**AQuAA (n = 48)**				
AQuAA Score (MET*min/wk)	5591 (3722;8786)	0.05	0.716	
Total physical activities (min/wk)	1330 (935;2344)			0.03 (-0.25 to 0.31)
Light-intensity activities (min/wk)	1063 (668;1898)			-0.001 (-0.28 to 0.28)
Moderate-to-vigorous intensity activities (min/wk)	238 (60;450)			0.32 (0.04 to 0.55)
Sedentary activities (min/wk)	3118 (2150;3720)			0.44 (0.18 to 0.64)
**PASE (n = 48)**				
PASE Sum score (PASE score)	87 (52; 162)	0.16	0.279	
Total physical activities (min/wk)	1609 (933; 2666)			0.12 (-0.17 to 0.39)
Sedentary activities (min/wk)	1079 (542; 1802)			0.39 (0.12 to 0.61)

AQuAA: Activity Questionnaire for Adults and Adolescents; CI: confidence interval; ICC: intraclass correlation coefficients; PASE: Physical Activity Scale for the Elderly; r_s_: Spearman's rho.

The Spearman correlation coefficient between the AQuAA score and the ActiGraph was low and not significant (r_s _= 0.05, p = 0.716). ICCs were poor for various PA scores (ICC = -0.001 to 0.32), and fair for sedentary activities; ICC = 0.44 (Table 3).

The correlations between the ActiGraph and the PASE were low and not significant (r_s _= 0.16, p = 0.279 and ICCs = 0.12 and 0.39) (Table 3).

The median time spent on physical activities was 1348 min/wk for the ActiGraph, 1330 min/wk for the AQuAA and 1609 min/wk for the PASE (see Table 3). 38% of the participants did not meet the American College of Sports Medicine (ACSM) recommendation of at least 150 minutes of moderate-intensity PA per week [36].

### Content Validity

Data saturation occurred after sixteen interviews. Several problems were identified during the first step of the interview (concurrent think aloud), which were clarified during the second step (focused interview), see Table 4. Patients perceived difficulties with the examples provided with the questions, had a different perception of the PA intensity level than stated in the questionnaires, or had difficulties with recalling the amount of time they had spent on PA. These difficulties did not seem to be age related, since it was reported by both the youngest and the eldest participant. Participants who indicated having no difficulties recalling the PA duration appeared to have a certain structure in their life which facilitated recall.

**Table 4:** Overview of cognitive difficulties identified during the Three-Step Test-Interview.

Issue	Difficulty	Questionnaire
Examples of activities were seen as an exclusive list	Not all of the examples provided to illustrate the questions were performed or the activities performed were not among the examples.	AQuAA and PASE.
	"*I climb the stairs, 7 times a day. I do not clean, but I do carry light loads. I do not know how to fill this out, so I will skip this*." (61 year old woman with non-Hodgkin's lymphoma).	

Different perception of intensity level.	The intensity level at which a certain activity was classified did not match the perceived intensity level.	AQuAA and PASE.
	"*Here it says that making the bed is a light household chore, but because I cannot do it, I find it a strenuous activity*." (47 year old woman with non-Hodgkin's lymphoma)	
	*"Jogging is classified as a vigorous intensity sport, however for me it is not more than a moderate intensity activity." *(26 year old woman with non-Hodgkin's lymphoma)	

Recall of frequency and duration.	Recalling the time spent on PA, other than sports and exercise, was challenging.	AQuAA.
	"*I do not constantly keep track of the time while cleaning the house*." (46 year old woman with cervical cancer)	

Calculating the amount of time spent on activities.	Whether the duration of physical activities should be divided across the actual number of days the activities were performed (e.g. 2 days) or across the whole week (i.e. 7 days).	AQuAA and PASE.
	*"If I perform an activity 2 days a week for 2 hours each day, to calculate the amount per week should I divide the total hours by 2 or 7 days?" *(59 year old woman with breast cancer)	

AQuAA: Activity Questionnaire for Adults and Adolescents; PASE: Physical Activity Scale for the Elderly.

In the last step (semi-structured interview), participants provided additional information regarding their experiences with the questionnaires (Table 5). Most participants indicated that they clearly understood the questions of both questionnaires, and that all daily PA were covered.

**Table 5:** Overview of the three themes and associated questions used to identify the participants' opinions of the questionnaires.

Theme	Typical answers provided by participants during the TSTI
**Interpretability**	*"Both the AQuAA and the PASE are clear to me, I understand all of the questions." *(34 year old man with non-Hodgkin's lymphoma)
Is the phrasing of the questionnaires clear to you?	*"I think that I was well capable of answering the questions of both questionnaires." *(50 year old woman with non-Hodgkin's lymphoma)
	*"The questionnaires are clear to me, nevertheless you have to take your time to read the questions well in order to answer them correctly." *(62 year old man with colon cancer)

**Comprehension**	*"All activities which people do in their daily life are asked for in the questionnaires." *(48 year old man with Hodgkin's lymphoma)
Are all (of your) daily physical activities addressed in the questionnaires?	*"Both questionnaires address all of my daily physical activities." *(50 year old man with non-Hodgkin's lymphoma)
	*"All of the activities I do in my daily life are included in the questionnaires." *(62 year old man with colon cancer)

**Preference**	"*I have always had trouble with focusing my attention, but now (after the cancer treatment) it has gotten worse...The PASE has multiple choice questions which is very helpful. This makes it easier for me*." (55 year old woman with breast cancer)
Which questionnaire were you more comfortable with answering?	*"The questions of the AQuAA are clearly arranged. It does however require more effort to fill in the AQuAA compared to the PASE because you really have to think about how much time you spent on the activities in the last week...The PASE is much easier because you can pick an answer which suits you best, you use your intuition to give an answer compared to the AQuAA where you really have to think...Therefore I prefer the PASE." *(48 year old man with Hodgkin's lymphoma)
	*"When answering the AQuAA I had to be very careful and it took a lot of effort for me to estimate how much time I spent on certain activities in the past week ...Because of this I prefer the PASE since you get an indication of the time you may have spent." *(66 year old woman with cervical cancer)
	*"You have to carefully read and think about the questions in the AQuAA, but it is not difficult to interpret...The PASE is much easier and because of the multiple choice questions you can be less precise in your answers, but this makes it somewhat superficial...Personally I prefer the AQuAA because I can identify myself more with the open questions structure." *(47 year old woman with Multiple myeloma)

AQuAA: Activity Questionnaire for Adults and Adolescents; PASE: Physical Activity Scale for the Elderly.

Thirteen participants preferred PASE, and three preferred the AQuAA. In general, the pre-structured answers of the PASE facilitated recall as opposed to the open-structure of the AQuAA.

## Discussion

This study evaluated the test-retest reliability, construct and content validity of the AQuAA and the PASE in cancer patients. Reliable and valid measures are needed to adequately assess PA levels in cancer patients. Improving PA levels of cancer patients is important in cancer rehabilitation as it may improve QoL and survival [5-7]. In our study we found that 38% of the participants did not meet the ACSM recommendation of at least 150 minutes of moderate-intensity PA per week. The median time spent on moderate-to-vigorous intensity PA in our study was 223 min/wk as measured with accelerometers. This is higher compared to previously reported studies of PA levels in breast cancer patients, which varied from 26 min/wk to 163 min/wk [13,14,24]. The higher PA level in our study may be related to younger age, a higher proportion of men, or other type of diagnoses (8% breast cancer).

### Test-retest Reliability

The test-retest reliability of the AQuAA and the PASE were good to excellent. The reliability of the AQuAA in the current study was higher compared to a previous study among healthy adults [17]. This may partly be caused by the shorter time interval between repeated administrations of the questionnaire in the current study (5 days versus 2 weeks). Alternatively, cancer patients may be more aware of their PA behaviour compared to healthy adults, resulting in better recall. The excellent test-retest reliability of the PASE sum score is in accordance with previously reported studies in healthy elderly [18,37].

We presented SEM and SDD_95 _values to indicate the magnitude of measurement error which should be taken into account when judging whether PA levels have really improved over time. Any increase in PA scores exceeding the SDD_95 _can be attributed, with reasonable confidence, to real improvements in PA level. High measurement errors were observed for both the AQuAA and the PASE. However, based on the calculated SDD_95_/range_95 _ratios we judge that the measurement error of both the PASE sum score and the time spent on total PA were sufficiently small (0.22 and 0.15 respectively) to make this questionnaire valuable for clinical practice. For these measurements the PASE is able to distinguish 5 and 7 steps on the observed measurement range. To detect change over time, distinguishing 7 steps (with a range of 5 to 9 steps) is considered adequate [30]. Final judgement about sensitivity to change requires a longitudinal follow-up study.

The good-to excellent reliability of sedentary activities indicate that the AQuAA and PASE might also be useful to assess sedentary behaviours.

### Construct Validity

The poor agreement between the ActiGraph accelerometer and the questionnaires do not confirm their construct validity. Previous studies of PA questionnaires in adults and the elderly also showed low correlations with accelerometers [31,32].

Our results showed low and nonsignificant correlations between the AQuAA and the ActiGraph for the total scores and the time spent on physical activities. These findings are in line with previously published reports of the AQuAA in healthy adults [17], and suggests, similarly to healthy adults [17], that cancer patients may also have difficulty with accurate recall of the duration and intensity of PA during the past 7 days.

In contrast to our findings, Dinger et al. [37] found a significant correlation between the ActiGraph and PASE (r_s_= 0.43; p < 0.01). The interview-based administration may have reduced over- or underreporting and misclassification of PA compared to the reliance on self-report in the current study. Otherwise, low correlations may result from the detection of light intensity PA by the accelerometer, while participants may not have realised to be physically active, and consequently did not report it. Furthermore, self-report questionnaires and accelerometers do not measure the exact same construct of PA. Accelerometers provide objective information on PA duration and intensity, whereas the AQuAA and the PASE also provide insight into the types of activities. Relatively high agreement between the accelerometer and sedentary scores assessed by questionnaire indicate their usefulness to assess sedentary behaviours.

### Content Validity

The TSTI method showed that participants perceived several problems when answering the AQuAA and the PASE regarding the type, intensity and duration of activities. This is an inherent problem of self-report questionnaires, and may also be associated with this particular study population due to their reduced fitness level and/or increased level of fatigue. Since the participants may perceive certain types of PA as more intensive or maybe compare their current PA level to their pre-diagnosis level, recall bias may have been introduced. This actually became clear during the TSTI, when several participants indicated certain activities to be more intensive than indicated in the questionnaire.

### Strengths and Limitations of the Study

This is the first study to combine both quantitative and qualitative methods to assess PA questionnaires in cancer patients. We extensively assessed the test-retest reliability, including measurement errors, and determined the construct and content validity of the questionnaires.

However, this study has several limitations. First, since there is no gold standard to measure PA, the accelerometer was used as comparison measure for the assessment of the construct validity of the questionnaires. However, both instruments have well-known limitations. Waist-worn accelerometers underestimate (light) upper-body movements, such as sweeping and weight-bearing activities, and other daily life activities such as swimming, bicycling and static activities [38]. Nevertheless, the accelerometer is technically speaking a reliable, precise and objective instrument [39]. Self-report measures of PA are limited by factors including social desirability, recall bias, and variations in cognitive and memory processes depending on several factors including age, education and gender [40]. These limitations may have contributed to the discrepancies observed between the ActiGraph accelerometer and the self-report questionnaires in this study. Second, standardized regression equations to calculate time spent on activities in different intensity levels by accelerometry are lacking. Accelerometer cut-off points for PA intensity and sedentary activities are still a matter of debate [41]. Although more recent cut-off points have been published [42], we chose to use the generally known and widely used Freedson cut-off points [23]. This allows for comparison of PA levels with other studies in cancer patients [13,14,24]. Researchers should take into account which regression equation and cut-off points were used when comparing studies, since the measured level of PA depends on the choice of cut-off points. Third, due to the broad duration categories, the PASE may not to be able to detect small changes in PA levels. Future studies are therefore needed to assess the responsiveness of the PASE.

## Conclusion

In conclusion, this is the first study providing insight in the psychometric properties of the AQuAA and the PASE in cancer patients using quantitative and qualitative methods. Test-retest reliability of both the AQuAA and the PASE were good to excellent for most scores. Based on the calculated SDD_95_/range_95 _ratios, we judge that the measurement error of the PASE sum score and time spent on total PA is sufficiently small to make it useful in clinical practice. Construct validity was low, but comparable to other PA self reports. Both questionnaires had good content validity. Most participants preferred the PASE because of its pre-structured questions.

## Competing interests

The authors declare that they have no competing interests.

## Authors' contributions

RDKL, LMB and MJMC participated in the design of the study, coordinated the data collection and performed the statistical analysis. MS and MJK coordinated the patient accrual. JB and WvM contributed intellectually to the draft of the manuscript. All authors read and approved the final manuscript.

## Pre-publication history

The pre-publication history for this paper can be accessed here:

http://www.biomedcentral.com/1471-2288/11/30/prepub
